# When procedures meet practice in community pharmacies: qualitative insights from pharmacists and pharmacy support staff

**DOI:** 10.1136/bmjopen-2015-010851

**Published:** 2016-06-06

**Authors:** Christian E L Thomas, Denham L Phipps, Darren M Ashcroft

**Affiliations:** 1 NIHR Greater Manchester Primary Care Patient Safety Translational Research Centre, The University of Manchester, Manchester, UK; 2 Manchester Pharmacy School, University of Manchester, Manchester Academic Health Sciences Centre (MAHSC), The University of Manchester, Manchester, UK

**Keywords:** Procedures, Patient Safety, Professional Autonomy, Community Pharmacy, Resilience

## Abstract

**Objectives:**

Our aim was to explore how members of community pharmacy staff perceive and experience the role of procedures within the workplace in community pharmacies.

**Setting:**

Community pharmacies in England and Wales.

**Participants:**

24 community pharmacy staff including pharmacists and pharmacy support staff were interviewed regarding their view of procedures in community pharmacy. Transcripts were analysed using thematic analysis.

**Results:**

3 main themes were identified. According to the ‘dissemination and creation of standard operating procedures’ theme, community pharmacy staff were required to follow a large amount of procedures as part of their work. At times, complying with all procedures was not possible. According to the ‘complying with procedures’ theme, there are several factors that influenced compliance with procedures, including work demands, the high workload and the social norm within the pharmacy. Lack of staff, pressure to hit targets and poor communication also affected how able staff felt to follow procedures. The third theme ‘procedural compliance versus using professional judgement’ highlighted tensions between the standardisation of practice and the professional autonomy of pharmacists. Pharmacists feared being unsupported by their employer for working outside of procedures, even when acting for patient benefit. Some support staff believed that strictly following procedures would keep patients and themselves safe. Dispensers described following the guidance of the pharmacist which sometimes meant working outside of procedures, but occasionally felt unable to voice concerns about not working to rule.

**Conclusions:**

Organisational resilience in community pharmacy was apparent and findings from this study should help to inform policymakers and practitioners regarding factors likely to influence the implementation of procedures in community pharmacy settings. Future research should focus on exploring community pharmacy employees' intentions and attitudes towards rule-breaking behaviour and the impact this may have on patient safety.

**Strengths and limitations of this study**

- This is the first study to specifically explore in depth opinions and experiences of working with procedures in community pharmacies.
- Detailed insights were provided by community pharmacists and support staff holding a range of roles and levels of experience from a variety of pharmacy settings.
- These qualitative findings highlight the importance of organisational factors in shaping how procedures are used in the context of community pharmacies.
- These findings should help to inform policymakers and pharmacy teams on how to optimise the implementation of procedures in practice.
- Future work is needed to further explore organisational resilience in community pharmacy, which should investigate the circumstances under which staff purposefully bypass or deviate from procedures and the potential impact this may have on patient safety.

## Introduction

A commonly encountered strategy for improving patient safety is the standardisation of healthcare practice, often by developing and implementing standardised procedures (in the form of guidelines, protocols and standard operating procedures (SOPs)).^1^ In principle, procedures provide assurance by holding healthcare staff to a minimum standard of practice and controlling aspects of their work that may create patient safety hazards.^2^ ^3^ However, the implementation of procedures has had a more limited effect on work practices than anticipated, with studies in hospitals,^4^ ^5^ general practices^6^ and community pharmacies^7^ finding that healthcare staff sometimes deviate from formal procedures in the course of their work.

Such findings have led researchers to examine the relationship between procedures and practice in healthcare. Reason *et al*^8^ and Dekker^9^ noted that strict adherence to inflexible procedures can make a task inefficient, or even unachievable, in practice. This is illustrated by the experience of one operating department, which found that adherence to a particular safety protocol interfered with the execution of surgical tasks.^10^ Other studies have identified a divergence between the nature of work assumed by formal procedures and that encountered in everyday practice; the latter being marked by varying and sometimes complex task demands, variation in the material and human resources available to achieve task goals, challenging work environments and diverse expectations on the part of staff members, patients, organisations and other stakeholders with regard to how task goals are defined and achieved.^11–15^

The relationship between procedures and practice can be understood in terms of organisational ‘resilience’—that is, the ability of an organisation or its members to maintain effective and efficient work in the face of a dynamic environment that is characterised by discontinuities in care, hazards, trade-offs and multiple goals.^16^ ^17^ According to the notion of resilience, staff may adapt their work activity in order to achieve task goals under the prevailing circumstances, thus creating a divergence between ‘work as imagined’ (as represented by the formal procedures) and ‘work as done’ (as represented by actual practice at a given time or in a given location).^9^ ^18^ ^19^ Hence, the effect of implementing procedures is determined by the relationship between these two aspects of work.

This study explores the use of procedures in England and Wales, and community pharmacy (CP) staff (in addition to their traditional role of supplying prescribed and non-prescription medicines) routinely provide advice on the management of minor ailments and the appropriate use of medicines, as well as conducting medicines usage reviews.^20–22^ Since 2005, pharmacies have been required to adopt standard operating procedures (SOPs) for the storage, dispensing and supply of medicines and the provision of medicines advice to patients.^23^ ^24^ A CP team can encompass a range of staff members including pharmacists, registered and non-registered support staff, medicine counter assistants and delivery drivers as well as a range of trainees who are all expected to abide by SOPs. However, evidence from studies of non-prescription medication supply, in the UK and in other countries, suggests that CP staff may not follow procedures as consistently as expected.^7^ ^25^ ^26^ Given the various demands and relationships that dictate the work of CP staff,^27^ ^28^ it is possible that the implementation of procedures in this setting is subject to interplay between formal expectations about how CPs should operate and how they operate in practice.^29^ The aim of the current study is to explore the experience of CP staff in applying procedures to their everyday work.

## Methods

### Design and setting

For the study design, we used semistructured interviews to collect qualitative data. The sampling frame for the study was CP staff in England and Wales.

### Sampling and recruitment

Participants were identified on a purposive basis, using departmental contacts, professional networks and advertisements on Twitter. One of the authors invited each of these participants to opt in to an interview about the use of procedures in their work via email. This recruitment was followed up with snowball sampling, in which the initial participants were asked to recommend other members of the sampling frame for the researchers to invite.^30–32^

Twenty-four participants (pharmacists (n=13), registered accuracy checking technician (ACT; n=1), registered technician (n=1), non-registered accuracy checking assistants (n=3) and dispensing assistants (n=6)) agreed to participate. These participants represented independent pharmacies (n=7), large pharmacy chains (n=9), medium-sized pharmacy chains (n=2), small-sized pharmacy chains (n=2), a supermarket (n=1) and locum/sessional staff (n=3; 2 pharmacists, 1 dispenser) who worked in a variety of pharmacy types. Participants worked in a range of locations including a city centre (n=1), a suburb (n=7), a town (n=10) and a village (n=2). Participants' time since qualifying in their role ranged from 6 months to 30 years. Participants' total time working in CP (either in their current role or in other roles) ranged from 2.5 to 35 years.

### Data collection

Semistructured interviews were conducted, focusing on the participants' opinions of procedures they are expected to follow in their role. The topic guide was developed from the literature on procedural compliance in healthcare and the first author's personal experience as a CP employee. Questions included:

- How are you made aware of the procedures that you need to follow during your work?
- How useful are procedures for helping you to do your job?
- Do you feel that you are able to follow the procedures at work?
- Are there certain times of the day, week, month or year that you feel procedures are typically deviated from or bypassed?

The topic guide was piloted with a member of CP support staff before data collection began. Interviews were conducted by the lead author between November 2014 and April 2015. Each interview was audio-recorded and transcribed in full. Interviews were held in a private place with only the participant and the interviewer present. Each participant gave informed written consent and interviews lasted 30–90 min. Participants were recruited until data saturation was reached and no new issues emerged during interviews.

### Analysis

Transcripts were analysed using a thematic analysis with a template method of organising qualitative data,^33^ and NVivo V.10 (QSR International) was used to support data analysis.^34^ A template of *a priori* thematic codes was created based on previous literature on compliance and views of procedures.^35^ The template was then independently applied to each transcript by the authors CELT and DLP (the latter not being involved in the interviews), who then discussed the coding and agreed on modifications to the template in order to represent the ideas identified in the data. Once the next version of template was agreed, it was refined through successive re-readings of the transcripts until no new themes emerged. The final template was then reviewed by DMA to ensure that it provided adequate coverage of the data.

## Results

Three main themes were identified, namely, the influence of work demands, the influence staff role has on how procedures are viewed and the dissemination and enforcement of SOPs. All participants appreciated the need for procedures in CP and agreed that the ultimate aim of procedures was to guarantee patient safety. Participants generally found SOPs useful for highlighting the ‘ideal’ way to work from a patient safety point of view. However, procedures were restrictive at times and could not be followed constantly for many reasons. Three main themes were identified: ‘the dissemination and creation of SOPs’, ‘complying with procedures’ and ‘procedural compliance versus using professional judgement’.

### The dissemination and creation of SOPs

One of the main themes with regard to the use of procedures as a whole in CP was how participants were made aware of the SOPs. Overall issues included the large amount of detailed procedures in CP. Participants felt complying with all procedures at all times was an unrealistic organisational aim given the complex setting and the high workload. The findings highlighted a difference in work-as-done and work-as-imagined due to the sometimes unrealistic expectations placed on CP staff, in terms of the large number of detailed procedures that resulted in difficulty for staff to learn and retain all of the procedures provided.

#### Dissemination of SOPs

Most participants were provided with written SOPs that they were expected to read on starting work in CP and this was often viewed as a prerequisite to start dispensing. Frequently participants mentioned an overload of procedures leading to difficulty in complying with expected practice. A pharmacist (P13) noted that procedures are ‘often left on a shelf and ignored’.

I couldn't dream of recalling every step of every policy and I don't think the staff that work with me could either…some might say that undermines the value of having all the rules because there's too many…but it's important that things are laid out.

(P1, Relief Pharmacist, Large Chain)

I think we often just read it, sign it and then you don't look at it again until you get told to…you never look anything up…

(P3, Dispenser, Large Chain)

[Staff are] presented with this massive folder [of procedures] and a lot of them are very repetitive…people will lose their attention span after five minutes…it defeats the point.

(P16, Locum Pharmacist)

I think the people who write the SOPs, they've never actually worked in [a branch] either.

(P12, Dispenser, Large Chain)

Disseminating pharmacy-specific SOPs to locum/sessional staff was noted as unrealistic, therefore making SOPs available to refer to when needed was important.

The agency I'm with have a lot of the [company] specific SOPs on their website…[So if] you've got a week in [a particular company], and they've got something particular that they do…you can read through before you go.

(P19, Locum Dispenser)

I think you'd be hard pressed to find a locum that could genuinely say, that if they walked into a pharmacy they'd never walked into before, they're going to spend an hour scouring the SOPs [before they start any work]…you can't work that way.

(P16, Locum Pharmacist)

#### The creation of SOPs

With regard to the creation of SOPs, the level of input from CP employees varied. A supermarket pharmacist (P20) noted that branch pharmacists are heavily involved in procedure development and that amendments were available for branches if needed. Having ‘frontline’ pharmacy staff comment on SOPs was useful for aligning work-as-imagined and work-as-done. Participants from an independent pharmacy spoke of the flexibility and control they had in creating and updating SOPs. Participants from a large pharmacy chain noted there was little flexibility and this could result in procedures that were not always appropriate.

Sometimes you can't follow them exactly…[they're] written for the whole of [the country] and each store…do things slightly differently even though they're all supposed to be the same. They try but they can't because customers want different things and surgeries do things differently…I think the company needs to recognise that they need to be a bit more flexible…

(P9, Pharmacist, Large Chain)

### Complying with procedures

A variety of factors affected compliance with procedures in CP. Participants from all roles emphasised the impact that work demands, workload and the behavioural norm within the team had on their ability to comply with procedures. Organisational factors were often attributed to result in a difference between work-as-imagined in the SOPs and the work-as-done by CP staff in practice.

#### Work demands

One of the main work demands that affected the ability of CP staff to comply with procedures was work scheduling, which was frequently mentioned by all staff types. Particular pressure points included public holidays and the beginning and end of the week. During these times, participants found complying with procedures challenging with some participants describing how working in CP on a weekend could feel like a ‘different job entirely’, mainly due to the closure of general practices and other resources not being available out of hours. Under these circumstances, pharmacists often resorted to applying their professional judgement regarding patient safety.

Easter weekend, the weekend before Christmas…the end of the week, Friday as well is usually very busy…sticking to the rules becomes less of a priority. [The job doesn't] become less of a priority, it's how you're doing the jobs…[it] depends on how much experience you have…you can [figure] out what you need to carry on doing by the rule book and what you don't.

(P10, Pharmacist, Large Chain)

Another crucial element that added to work demands was staffing levels. Participants in all roles expressed how following procedures were especially difficult with insufficient staff for their pharmacy.

Staffing and [lack of] time are probably the biggest things that put extra pressure on what you're doing, and maybe lead to [some things] not quite going as they should do.

(P19, Dispenser, Large Chain)

#### Workload

Many participants spoke of regularly attempting to complete several tasks at once to manage workload, leading to occasional shortcuts. All participants mentioned the volume of tasks they had to complete under time pressure. Pharmacists also highlighted the need to achieve service targets set by head office or area management regarding professional services such as medicines usage reviews^21^ and the new medicine service.^22^

…The number of items goes up every year, the time [you have] to spend just doing those goes up and up and more and more services come out at the same time. [The challenge is] having time with the patient to do everything you can for them, so [following the] rules comes into that and it's really hard [to manage].

(P15, Pharmacist, Large Chain)

The general thing [is] time…either you have too much work or…your colleagues isn't there…there's always steps in the SOPs which you cannot do, but still get the same result at the end…

(P3, Dispenser, Large Chain)

#### Behavioural norms

Participants often spoke of the ‘the way we do things around here’, which did not always coincide with work-as-imagined in SOPs. Sessional pharmacists and support staff felt under pressure to conform to local practice, even if this was not outlined in procedures. This resulted in differences between branches of the same company, despite an apparent purpose of SOPs being to standardise performance.

…most of the time you just end up shutting up that side of you that's saying… don't do that, and [instead] you say if that's what [the regular pharmacist does] then I'll just do the same…you might be not 100 per cent sure of what's going on…because of the demand around you [from the support staff]…you just get on with it.”

(P11, Locum Pharmacist)

It would be nice to get a bit more back-up from pharmacists [regarding following the procedures]…the [procedures are] not just there for one person, they're there for everyone and it's safer if everybody follows the [procedures] properly.

(P22, Accuracy Checking Dispenser, Independent Pharmacy)

### Procedural compliance versus using professional judgement

There were varied opinions between participants about the relative merits of standardised practice and the use of professional judgement by CP staff. In our sample, the variation in opinion was particularly noticeable when comparing the views of the pharmacists with those of staff in other roles. The pharmacists appreciated that procedures were useful to an extent, and also felt that they reserved the right to bypass or deviate from procedures if they judged it necessary for the patient's outcome.

There are scenarios where the patient's health is at risk if you follow them. So sometimes, you do have to make your own decision on what is best for the patient's care, because that's the most important thing to do as a pharmacist.

(P4, Pharmacist, Large Chain)

If somebody's on their way to dying and the doctor's forgotten the Midazolam CD schedule three, and forgot to put the quantity, where the figures all look clear and [the prescriber says] ‘okay, we're on visits, we'll be over in an hour to sign it’. Do I leave it an hour? The patient could be dead in an hour.

(P6, Pharmacist, Independent)

However, some participants expressed concern that acting outside of procedures exposed them to the risk of disciplinary action or litigation.

I think if something's gone wrong then I'd definitely go back and have a look at the SOPs…[unless] you're the actual pharmacy manager there and you work there full-time, you [don't] have the time to take [SOPs] home [to read]…

(P16, Locum Pharmacist)

I suppose people follow the bits they agree with and they don't follow the bits they don't agree with. And being in a big company, there's not really a lot you can do about the bits you don't agree with. It's not like they're going to change it, so you just have to take it upon yourself, which then leaves you open to being uninsured if you don't follow them, so it's a lose-lose situation really, but everybody kind of does it.

(P9, Pharmacist, Large Chain)

There were some procedures that were considered important enough that participants would adhere to them even in unfavourable circumstances.

[Bypassing the procedure that states I should not work in the pharmacy alone means I can] actually do things properly. There are certain things that I would never be happy cutting corners with. I want to do a full CD balance every week. I'm not going to not do that. So if that means doing overtime for free then I'm going to do it…it's protecting myself, it's protecting my registration…

(P14, Store-Based Pharmacist, Large Chain)

Interestingly, newly qualified pharmacists seemed to rely on SOPs as a guide to practice; however, more experienced pharmacists noted that this was not a realistic approach to professional practice.

When you newly qualify…you've literally swallowed up the [Medicines, Ethics and Practice professional guide for pharmacists] and you're so into the laws that when it comes to practise it's quite shocking how much deviation takes place in a pharmacy …I was extremely cautious and very worried and I'd go home and I'd start thinking about everything that had happened [at work]. But, then eventually…you get used to it.

(P11, Locum Pharmacist)

I think [making a professional decision] scares some pharmacists, some of them want it in black and white…pharmacy can't be black and white. But that's why we are professionals because we make those decisions. Anyone can follow a process, a dispenser can follow a process…the pharmacist has to make a professional decision.

(P20, Pharmacist, Supermarket)

At times pharmacists would face situations in which there was no set guidance and professional judgement was crucial.

There's a balance…I think the trouble with our profession is that we want a rule for everything and that's not how a profession works…We shouldn't anticipate that there's always going to be an SOP for everything.

(P13, Pharmacist, Medium-Sized Chain)

All participants allude to ‘professionalism’—for pharmacists though, professionalism is about exercising professional judgement, whereas for some support staff, it was about following rules. For support staff, professional judgement plays less of a part in their role—so following procedures was seen as a way to ensure patient safety.

SOP's are in place to make sure that we're doing the right thing… If we don't do as we're told when we're dispensing, then it's a danger to the patient.

(P22, Non-Registered Accuracy Checker, Independent Pharmacy)

I don't want to be struck off…sticking within the rules, makes sure that the patient's safe. Go out of the rules and the patient's not safe, and neither's your job.

(P24, Registered ACT, Independent Pharmacy)

Being registered with the GPhC has a huge influence on the way that I feel, because I want to keep it…I value my job and I do value the rules…because I'm registered, I think it heightens my realisation that there are rules because I am responsible for myself and my own actions…

(P24, Registered ACT, Independent Pharmacy)

Notably, dispensers' attitude towards procedures was seen to be more flexible at times. Some felt procedures were a ‘tick box exercise’ and did not necessarily shape their work to a large extent. Although dispensers are required to sign to say they have read and will abide by SOPs, in certain circumstances, the instructions of the responsible pharmacist were followed as an alternative. Dispensers especially did not always feel that they are able to question the decision of the responsible pharmacist.

[I do] what the pharmacist is telling me to do, because they're responsible for what goes on, so it's their call.

(P18, Relief Dispenser, Large Chain)

## Discussion

The participants saw procedures in CP to be useful for setting expectations of practice and for improving knowledge, yet tensions were evident between the standardisation of practice and the scope of behaviour available to pharmacy staff in completing their tasks. The need to deviate from work-as-imagined when patient safety was at risk was an important part of being a professional for pharmacists. Dekker^9^ describes tension between procedures and safety as a considerable practical problem. A successful outcome for patient safety is not guaranteed from following procedures but created from a diversity of responses that allow staff to cope with their changing environment.^15^

Our findings expose elements of organisational resilience in CP. Participants relied on their ability to adjust, dealing with standardised systems on the one hand and with non-standardised situations on the other. This flexibility is fundamental to working in CP, as employees create changes to procedures and accommodate changes in order to meet patient needs.^36^ The formation of rule-related behavioural intentions in CP could be compared with the findings of Phipps and Parker,^37^ which found anaesthetists sometimes worked ‘in the moment’ when deciding how to act in a given situation. This process is most likely to occur in settings such as CP, as it involves a multidisciplinary team, time pressure, emergency situations, shifting goals, organisational norms and goals that may go against the employee's interests.^15^ ^38^ Phipps and Parker^37^ note that these are areas where procedural violations should be of ‘most concern’. Evidence of procedural violations in CP is limited,^7^ ^39^ and further exploration of this topic is suggested to assess potential risk to patient safety.

We found some variations in the views of participants about procedures. Some of these variations might be attributed to differences in role and responsibility between participants. For example, pharmacists tended to express similar attitudes to doctors and surgeons in previously published studies that identified the need for a degree of flexibility was required when working in healthcare as oppose to the notion of ‘cookbook’ care.^12^ ^13^ ^40^ The pharmacists here appeared to invoke the notion of professional autonomy with regard to following procedures, echoing previous research exploring implementation of emergency hormonal contraception services in community pharmacies.^41^ The attitudes of registered support staff were similar to nursing staff, as they approached patient safety by systematically following procedures.^42^ ^43^ In contrast, dispensers had a more flexible approach, as the ultimate responsibility for their actions was that of the responsible pharmacist at the time.^44^ However, when following instructions that do not benefit the patient, we identified difficulties of support staff not feeling able to voice concerns.^13^ ^45–47^ Failing to communicate has been identified as a key threat to patient safety.^48^ Therefore, a culture in which all CP employees feel that they are able to discuss adherence to or deviation from procedures needs to be encouraged.

Adherence to procedures may help manage risks to patient safety in some circumstances; an overreliance on procedures could be counterproductive. Efforts to align work-as-imagined with work-as-done would be beneficial for creating SOPs that are more reflective of practice while providing an effective risk control. One method for achieving this is to maintain a dialogue between ‘frontline’ staff and those responsible for creating SOPs regarding the correspondence between the SOPs and actual practice.

### Study limitations

Though our study is the first to explore the experience of CP staff in applying procedures to their everyday work, it has some limitations. A limitation of this study is that all members of a CP team were not invited to participate. Healthcare counter staff were excluded as previous research has already focused on how procedures are followed with over the counter (OTC) medicines,^7^ and our aim was to focus on the dispensary due to the large number of existing procedures relating to the safe supply of prescribed medicines.

A limitation could also be the inclusion of senior pharmacists (whose roles included setting and disseminating SOPs) as their opinions may differ due to decreased time practicing. However, it was thought that their experience and knowledge of procedures in CP would help to enrich our understanding. Therefore, future work exploring the suggested differences in opinion of CP staff on a larger scale would be beneficial with regard to how employees are trained.

It should be noted that the topic guide would have affected on the data collected, as will the use of *a priori* themes. In an effort to account for this, semistructured interviews were undertaken to provide participants with the opportunity to discuss issues they believed to be salient to the use of procedures in CP that may not have been captured within the topic guide. To account for any potential bias caused by the interviewer's personal experience of working in CP, the data were triangulated between all authors during data analysis.

### Study implications

#### Theoretical implications

This study supports the use of organisational resilience as a valuable concept for understanding how procedures are viewed and used in CP. The notion of resilience helps to explain how CP staff attempt to manage multiple goals, while coping with high workloads and low staffing in an effort to ensure patient safety. It is thought that the concept of organisational resilience would be useful to further explore specific instances of CP staff bypassing or deviating from procedures.

#### Implications for practice

The findings from this study have important implications for pharmacy practice and policy, as they highlight the role that organisational factors have on how procedures are implemented in CP. Factors such as pressure from the organisation regarding achieving targets or setting procedures that are difficult to follow when working out of hours can create an environment where following procedures and achieving required outputs is sometimes felt to be unachievable. Furthermore, the fear of being unsupported by an employer if procedures were not complied with, even if it was for patient benefit, created an additional pressure to pharmacists.

On the other hand, this study has shown that the use of professional judgement is crucial when deciding whether to comply with a procedure. A suggested implication for practice is the notion of an appropriate and justified flexibility, allowing the responsible pharmacist to make professional judgements with the support of their employer in order to ensure patient safety. The aim of this article is not to undermine the important role that procedures play in CP, but our results suggest that there are times in which bypassing or deviating from procedures may be required for patient safety.

### Suggestions for future research

Future work is needed to investigate instances in which CP staff deviate or bypass procedures and how organisational factors noted in this study may have contributed to the decision to do so. In addition, based on this study, further work exploring the attitudes of CP staff and their intention to follow procedures on a larger scale may have important implications for patient safety.

## Conclusion

This study examines how procedures are viewed by staff in community pharmacies and how this can affect professional autonomy. The findings highlight the tension between standardising practice and the need, at times, for greater flexibility for pharmacists to decide on the most appropriate course of action to manage risks to patient safety. Evidence of organisational resilience in CP practice was apparent and the findings should help to inform policymakers and practitioners with regard to the factors most likely to influence the implementation of procedures in CP. We suggest more work is needed in practice to ‘realign’ work-as-imagined and work-as-done; one suggestion is to improve communication between staff on the ‘frontline’ and management to lessen the gap between the two.^19^
